# Enhancing the therapeutic impact of sublethal radiofrequency hyperthermia in malignant solid tumor treatment

**DOI:** 10.1016/j.heliyon.2024.e29866

**Published:** 2024-04-17

**Authors:** Jiayun Liu, Guilin Zhang, Xinyi Li, Chuansheng Zheng, Xuefeng Kan

**Affiliations:** a Department of Radiology, Union Hospital, Tongji Medical College, Huazhong University of Science and Technology, Wuhan 430022, China; b Hubei Province Key Laboratory of Molecular Imaging, Wuhan 430022, China

**Keywords:** Radiofrequency ablation, Radiofrequency hyperthermia, Chemotherapy, Radiotherapy, Immunotherapy, Gene therapy

## Abstract

Radiofrequency ablation (RFA) is an effective alternative to surgery for managing some malignant solid tumors. However, for medium-to-large tumors (>3 cm), tumors adjacent to large blood vessels, and certain irregular tumors, sublethal radiofrequency hyperthermia (RFH) often produces a margin of ablated tumor owing to the “heat-sink" effect. This effect typically leaves behind viable residual tumors at the margin. Several studies have reported that a sublethal RFH can significantly enhance the efficacy of chemotherapy, radiotherapy, immunotherapy, and gene therapy for malignant solid tumors. The possible mechanisms by which RFH enhances these therapies include heat-induced tissue fracturing, increased permeability of the cytoplasmic membrane, exaggerated cellular metabolism, blockade of the repair pathways of radiation-damaged tumor cells, and activation of the heat shock protein pathways. Therefore, RFA in combination with chemotherapy, radiotherapy, immunotherapy, or gene therapy may help reduce the rates of residual and recurrent tumors after RFA of malignant solid tumors.

## 1 Introduction

**Fig. 1:**
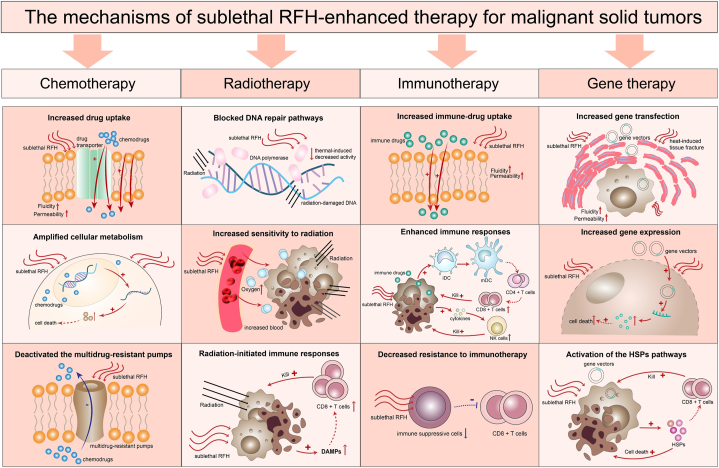
The mechanisms of sublethal RFH-enhanced chemotherapy, radiotherapy, immunotherapy, and gene therapy for malignant solid tumor. For chemotherapy, the sublethal RFH enhanced-mechanisms included modulating the function of the cPt transporter Ctr1, amplifying the cellular metabolism, and deactivated the multidrug-resistant pumps. For radiotherapy, the sublethal RFH enhanced the efficacy of radiotherapy by blocking the DNA repair pathways, increasing the sensitivity to radiation, and initiating the radiation-related immune responses. For immunotherapy, the sublethal RFH enhanced the effect of immunotherapy by increasing the accumulation of the immunotherapeutics, improving the immune response to tumor cells, and reducing the resistance to immunotherapy. For gene therapy, the sublethal RFH enhanced mechanisms involved in increased gene transfection, up-regulated gene expression, and activation of the heat shock protein pathways.

Radiofrequency ablation (RFA) is an effective alternative to surgery for the management of some early-stage solid malignancies, such as hepatocellular carcinoma (HCC), lung cancers, and renal carcinoma [[1], [2], [3]]. However, the incidence of post-RFA tumor recurrence remains relatively high at the ablation margin, particularly in medium-to-large tumors (>3 cm), tumors adjacent to large blood vessels, and certain irregular tumors [4,5]. According to the literatures, the approximate rates of local tumor recurrence in patients with HCC, lung cancers, or small renal carcinoma treated by RFA were 59.8%–63.1 % [6], 26 % [7], or 17.5 % [8], respectively. Incomplete RFA of tumors poses a major challenge in creating a safe and effective ablation periphery of a 1-cm surgical margin beyond tumor confinement. Failure to achieve this clear margin is usually caused by the “heat-sink” effect, i.e., the thermal ablation heat is carried away by blood flow when the target tumor is located near large or major vessels. This in turn leads to sublethal radiofrequency hyperthermia (RFH) at the ablated tumor margin during RFA procedure. Residual tumors stimulated by sublethal RFH at the ablated tumor margin tend to be the source of tumor recurrence and thus progress swiftly [9,10]. In addition, several previous studies [[11], [12], [13]] [[11], [12], [13]] [[11], [12], [13]] reported that a sublethal RFH could promote tumor proliferation and progression. However, in recent years, treatment strategies that combine combined sublethal RFH with other therapies, such as chemotherapy, radiotherapy, immunotherapy, and gene therapy, have been developed to treat malignant solid tumors because RFH can enhance the treatment effects of these therapies [14,15]. In this study, we conducted a comprehensive review of sublethal RFH enhancing these therapies for the management of malignant solid tumors (Fig. 1), with the aim of summarizing the treatment strategies that reduce the post-RFA residues and recurrence of ablated tumors.

## 2 Methods

### 2.1 Search strategy

The preferred reporting items for systematic reviews and meta-Analyses (PRISMA-2020) guideline was applied to standardize the literature search of this review. We systematically searched the Pubmed, Embase, and Cochrane Library for relevant articles published from January 1, 2010 to October 31, 2023. The search strategy was on the basis of following Mesh terms: “radiofrequency hyperthermia”, “insufficient radiofrequency ablation”, “incomplete radiofrequency ablation”, “chemotherapy”, “radiotherapy”, “immunotherapy”, “gene therapy”, “cancer therapy”, “malignant solid tumor”, “hepatocellular carcinoma”, “lung cancers”, “renal carcinoma”, “cholangiocarcinoma”, “non-muscle invasive bladder cancer” and “cervical cancer”. The “AND” and “OR” boolean operators were used individually or in combination to perform multiple keyword searches. No language restriction was applied. We also reviewed the reference lists of all relevant articles to identify additional studies.

### 2.2 Eligibility criteria

**Fig. 2:**
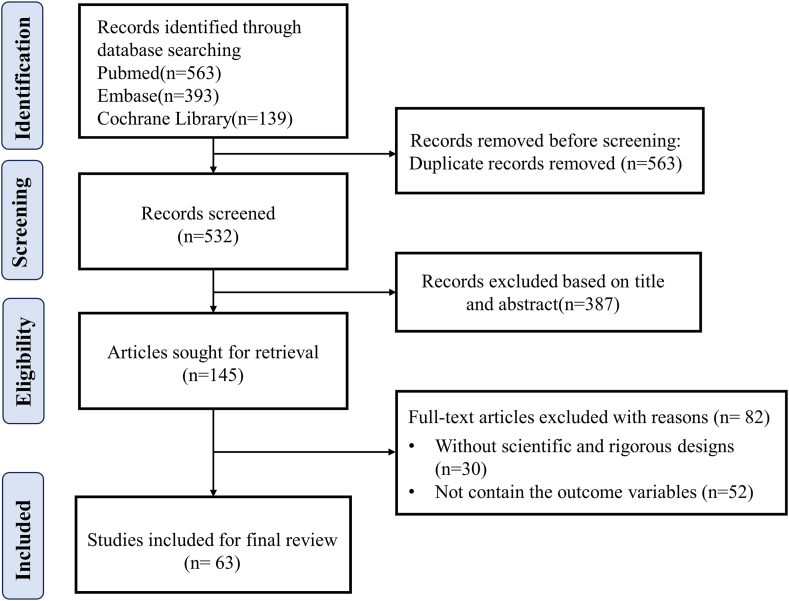
The flow diagram of study selection.

The articles about sublethal RFH in combination with other therapies for malignant solid tumors were included in this review, which included the clinical and preclinical studies. We also included gray literature (ie, materials and research produced by organizations outside of the traditional commercial or academic publishing and distribution channels, such as reports, working papers, and evaluations). Studies without scientific and rigorous designs or a quantitative efficacy evaluation for the treatments of malignant solid tumors were not included in this review (Fig. 2).

## 3 Sublethal RFH enhanced chemotherapy for malignant solid tumors

Previous studies [[16], [17], [18], [19]] have demonstrated that the hyperthermia (42 °C) in the tumor tissues could increase the fluidity of the bilayer of phospholipids of the tumor cells and the permeability of the cytoplasmic membrane, promote the metabolism of tumor cells, deactivate the multidrug-resistant pumps, and modulate the cPt transporter Ctr1, thereby facilitating the entry of chemotherapeutic agents into the tumor cells. These findings suggest that, mechanistically, sublethal RFH could significantly enhance the effect of chemotherapeutics on some malignant solid tumors, and that sublethal RFH in combination with some chemotherapeutic regimens might exert a synergistic anti-tumor effect.

In recent years, Yang et al. examined the enhancing effect of RFH on chemotherapies for liver cancers [14,15,20,21]. They established animal models of orthotopic or subcutaneous liver cancers, and developed a multimodal perfusion-thermal RFA electrode, which allowed for the simultaneous peritumoral infusion of chemotherapeutic agents and application of the radiofrequency hyperthermia. The electrode was placed into the tumors with its prongs covering the entire tumor under ultrasound guidance. The therapeutics were infused into the tumor via the prongs of electrode, followed by intratumoral RFH at 42 °C lasting for 30 min administered to the liver cancers. They found that this sublethal RFH could significantly enhance the efficacy of doxorubicin on VX2 liver tumors and the effect of liposomal doxorubicin on HCC, thereby providing a new treatment alternative for lowering the rates of residual lesions and recurrence after RFA in large liver cancers.

Although systemic chemotherapy is one of the main treatment strategies for pancreatic cancer, cholangiocarcinoma, breast cancer, esophageal cancer, and prostate cancer, the results in these patients remained unsatisfactory in terms of efficacy and toxic side effects. The potential main reasons include: (1) the insufficient dose of chemotherapeutic drugs delivered to the targeted tumor, which resulted in limited efficacy and increased dose-related toxicity for patients; and (2)inadequate deposition of chemotherapeutic agents in the target tumors via systemic administration, which induced the development of chemoresistance [22].

To address these issues, in several studies [[23], [24], [25], [26], [27]], the chemotherapeutics were directly injected into the tumor through puncture, and the 30-min RFH at about 42 °C was performed by inserting an imaging heating guidewire into the tumors via an injection needle immediately after the drug delivery. This strategy effectively overcame the disadvantages of the systemic delivery. The results showed that this sublethal RFH significantly improved the effect of gemcitabine on pancreatic cancer, gemcitabine and 5-FU on cholangiocarcinoma, doxorubicin on breast cancer, cisplatin and 5-fluorouracil on esophageal cancer, and docetaxel on prostate cancer, thus providing novel treatment options for malignant solid tumors and paving the way to the further studies on their future clinical applications.

Non-small cell lung cancer (NSCLC) is the most common type of lung cancers [28]. The regimen of gemcitabine and cisplatin (GC) is widely employed as the first-line treatment for advanced NSCLC [29]. Nonetheless, the efficacy of this regimen is far from satisfactory for treating advanced NSCLC. A phase Ⅱ clinical study [30] used a HY-7000 radiofrequency external heat system to achieve the regional/local hyperthermia. The region of the target primary lung cancer was covered by an applicator 150–300 mm in diameter, and a sublethal RFH at 39°C–42.5 °C was applied 1 h after intravenous administration of GC. The results showed that this strategy significantly enhanced the response of NSCLC to therapy compared to GC alone. Meanwhile, the combined use of RFH significantly improved the patients’ quality of life. This study suggested that sublethal RFH could serve as a complementary and effective modality in the multidisciplinary treatments of advanced NSCLC.

Bladder cancer is the fifth most frequently diagnosed cancers in Northern America and Europe, and most of bladder cancer patients have non-muscle invasive bladder cancer (NMIBC) [31]. The recommended treatment for NMIBC is a transurethral resection of the bladder tumor, with or without adjuvant intravesical instillation of Bacillus Calmette-Guérin or chemotherapeutic agents, including epirubicin or mitomycin-C [32]. However, even with intravesical medications, the recurrence rate of NMIBC remains high [33]. Owing to the high risks of mortality and morbidity, NMIBC patients are unwilling or frequently unfit to undergo radical cystectomy when standard intravesical treatment fails. For these patients, several prior studies [32,34,35] delivered/applied a sublethal RFH at 40–43 °C to the bladder wall, by placing a radiofrequency applicator in the bladder via a urinary catheter, in combination with circulation of mitomycin-C or epirubicin in the bladder and yielded encouraging results. Furthermore, a randomized controlled trial [36] compared chemohyperthermia using sublethal RFH and mitomycin C with the bacillus Calmette-Guérin as an adjuvant for intermediate- and high-risk NMIBC, and achieved a significantly higher 24-month recurrence-free survival in the chemohyperthermia group than in the bacillus Calmette-Guérin group (81.8 % vs. 64.8 %, p = 0.02). Taken together, these results demonstrated that the chemohyperthermia integrating RFH and mitomycin C or epirubicin may be an optimal treatment option for intermediate- or high-risk NMIBC patients.

## 4 Sublethal RFH enhanced radiotherapy for malignant solid tumors

The mechanisms by which sublethal RFH and radiotherapy work synergistically in malignant solid tumors are as follows. First, the tumor-killing effects of RFH and that of radiotherapy complement each other. Generally, tumor cells in the S phase of the cell cycle are less sensitive to radiotherapy but respond relatively better to hyperthermia. The sensitivity of anoxic tumor cells to radiotherapy is lower, but that to hyperthermia is relatively stable [37,38]. Second, sublethal RFH can increase the oxygen partial pressure and blood flow in treated tumors, thereby effectively improving the oxygen supply to tumor cells, and subsequently leading to a significantly enhanced radiotherapeutic effect on some malignant tumors [39,40]. Third, sublethal RFH can block the repair pathways of radiation-damaged tumor cells by decreasing the activity of DNA polymerase, which could effectively inhibit the repair of DNA single-strand breaks caused by radiotherapy [41,42]. Fourth, sublethal RFH can trigger a systemic immune response during radiotherapy and enhance its anti-tumor efficacy of radiotherapy [43]. Presumably, sublethal RFH combined with radiotherapy for the treatment of some malignant solid tumors is based on these mechanisms.

In recent years, mounting evidence [[44], [45], [46], [47]] suggested that radiotherapy is effective and safe for HCC, and that radiotherapy combined with transarterial chemoembolisation or immunotherapy are promising alternatives for unresectable HCC. In 2016, Dong et al. [48], in a randomly control clinical study, compared the conformal radiotherapy plus RFH with conformal radiotherapy for advanced HCC patients. Initiating from the first day of radiotherapy, they non-invasively performed a sublethal RFH at about 41 °C twice a week. Their results showed that RFH in combination with conformal radiotherapy significantly decreased the 6-month and 1-year recurrence and mortality rates (p < 0.001) compared to those of radiotherapy alone. Moreover, they found that the sublethal RFH could improve the efficacy of radiotherapy for advanced HCC, and function as an enhancer in the radiotherapy-based comprehensive treatment of HCC.

Radiotherapy is a major treatment option for early stage unresectable NSCLC [49]. Although stereotactic body radiation therapy is effective for these tumors, long-term follow-up has shown that local recurrence and distant metastasis often occur, suggesting that the efficacy of radiotherapy for unresectable NSCLC requires further improvement [49]. Several previous studies [[50], [51], [52]] indicated that the sublethal RFH could improve the radiotherapy for unresectable NSCLC, and could be used to improve the local tumor control for NSCLC patients receiving radiotherapy. These results suggested that the sublethal RFH plays a significant role in the comprehensive treatment of NSCLC with radiotherapy.

Radiotherapy is an integral component of multimodal treatments for breast cancer [53]. Previous studies have also explored whether the sublethal RFH can enhance radiotherapy for breast cancer. Mohammadi et al. [54] used a clonogenic assay to investigate the radiosensitizing effect of RFH in combination with PEGylated gold nanoparticles on MCF-7 breast cancer cells under electron beam radiotherapy, and found that a low nontoxic concentration of 20 nm PEG-GNPs significantly increased the radio-sensitizing effect of RFH plus 6-MeV electron beam radiotherapy on MCF-7 breast cancer cells. Hehr et al. [55] assessed the effectiveness of a sublethal RFH in combination with radiation therapy for locally recurrent breast cancer after primary modified radical mastectomy. In their study, the sublethal RFH at about 41 °C was administered twice a week immediate before radiotherapy. The results showed that the median overall survival time was 28 months, with 71 % of the patients living for 1 year and 54 % living for 2 years after the thermoradiotherapy. These results indicate that the sublethal RFH can enhance the radiotherapeutic effects in breast cancer.

Cervical cancer is one of the most common malignancies and a leading cause of cancer-related deaths in women [56]. According to the International Federation of Gynecology and Obstetrics (FIGO) for stage III/IV cervical cancer, the mainstay treatment is radical radiotherapy alone, and the 5-year survival rate is approximately 30 %–40 % [56]. Harima et al. [57] conducted a randomized clinical trial that compared radiotherapy alone with RFH plus radiotherapy for FIGO stage IIIB cervical carcinoma, and found that the complete response rate was significantly higher in the combination radiotherapy group than in the radiotherapy-alone group (80 % vs 50 %, p = 0.048); additionally, the 3-year local relapse-free survival rate was significantly higher in the combination group (79.7 %) than in the radiotherapy-alone group (48.5 %) (p = 0.048). These results demonstrate that the sublethal RFH in combination with radiotherapy might lead to a better treatment effect for FIGO stage IIIB cervical carcinoma than radiotherapy alone.

## 5 Sublethal RFH enhanced immunotherapy for malignant solid tumors

Previous studies [[58], [59], [60]] have demonstrated that hyperthermia could modulate the tumor immune microenvironment by eliciting danger signals through heat shock proteins, subsequently activating the immune systems, increasing the concentrations of some anti-tumor immunotherapeutic agents in tumor cells, and suppressing the resistance of some malignant solid tumors to the agents. These findings have led to the exploration of the potential to enhance the anti-tumor effects on certain malignant solid tumors by combining RFH and immunotherapies.

Oncolytic immunotherapy is a promising treatment option for some solid malignant tumors. LTX-315 or LTX-401, a first-in-class oncolytic peptide, can kill cancer cells by exerting a membranolytic effect on the cellular membrane and intracellular organelles, such as Golgi complexes and mitochondria, which leads to the subsequent release of danger-associated molecular pattern molecules and tumor antigens that recruit and activate T cells essential for immunotherapy [61,62]. Talimogene laherparepvec (T-VEC), a herpes simplex virus-1 (HSV-1)-derived and genetically-modified oncolytic virus, selectively replicates viruses within the neoplastic cells, resulting in a direct lytic effect on tumor cells and the activation of systemic anti-tumor immunity [63]. Several studies [14,[64], [65], [66], [67]] found a sublethal RFH at 42 °C could significantly enhance the anti-cancer effects of LTX-315, LTX-401, and T-VEC on hepatic cancers or pancreatic adenocarcinoma through an increase in CD8+T cells, CD8^+^/IFN-γ^+^ T cells, CD8^+^/TNF-α^+^ T cells, and NK cells, along with a reduction in Tregs in the tumor microenvironment, thus achieving an optimal treatment effect. These studies suggested that a sublethal RFH combined with oncolytic immunotherapy was a promising treatment strategy for some malignant solid tumors.

Treatment with cytokine-induced killer cells represents a new practical option for cancer immunotherapies, and is a feasible and effective adoptive immunotherapy for the treatment of solid tumors [68]. Wang et al. [69] treated 31 advanced HCC patients using intraperitoneal perfusion of cytokine-induced killer cells combined with sublethal RFH. They found that the serum levels of CD4^+^, CD3^+^, CD8^+^, and CD3^+^CD56^+^ cells significantly increased after the combination treatment, with a disease control rate of 67.7 %. However, this study lacked power because it did not include a control group, i.e., the patients receiving cytokine-induced killer cells. Vancsik et al. [70] used a mouse xenograft model of human melanoma to test RFH (∼42 °C) both alone and in combination with NK-cell immunotherapy. They found that when RFH was administered with either primary human NK cells or the IL-2-independent NK-92MI cell line injected subcutaneously, NK cells accumulated in the RFH-pretreated melanoma nodules but not in the untreated controls. Sublethal RFH significantly enhanced intratumoral recruitment of distantly injected NK-cells, induced by CXCL11 and MMP2 upregulation, resulting in an additive effect on growth inhibition and tumor destruction. Sublethal RFH may serve as an effective adjuvant treatment for cytokine-induced killer cell therapy in malignant solid tumors.

Immune checkpoint inhibitors, such as PD-1, PD-L1, and CTLA-4 inhibitors, showed promising therapeutic effects against some malignant solid tumors. However, monotherapy involving a single immune checkpoint inhibitor usually resulted in low tumor response [71,72]. Multiple studies [[73], [74], [75]] reported that incomplete RFA of malignant solid tumors could promote rapid progression of the residual tumors towing to the stimulation of sublethal RFH. Zhang et al. [76] created a mouse model of incomplete RFA subcutaneous HCC, and evaluated the effects of CTLA-4 therapy on residual tumors. They found that the anti-CTLA-4 therapy could significantly suppress the growth of residual tumors with the level of CD4^+^ T cells, CD8^+^ T cells, and IFN-γ being significantly higher in the residual tumors than in the non-treated tumors. These results indicated that the sublethal RFH, in combination with immune checkpoint inhibitors, has potential as an alternative for certain malignant solid tumors.

## 6 Sublethal RFH enhanced gene therapy for malignant solid tumors

Gene therapy is another treatment option for certain cancers. The possible mechanisms of RFH-enhancing gene therapies include heat-induced tissue fracturing, increased permeability of the cytoplasmic membrane, activation of heat shock protein pathways, and elevated cellular metabolism [17]. These mechanisms facilitated the entry of gene therapeutics into target tumor cells, thereby effectively promoting tumor destruction.

Among various oncological gene therapies, the most widely accepted is tumor suicide gene therapy, also known as the herpes simplex virus-thymidine kinase (HSV-TK)/ganciclovir (GCV) therapy. Effective HSV-TK/GCV gene therapy depends on sufficient transfection of HSV-TK genes into tumor cells, where it can convert non-toxic GCV to highly toxic phosphorylated GCV. In recent years, a series studies by Yang et al. showed that a direct intra-tumoral injection of high-dose HSV-TK/lentivirus genes, via a multifunctional thermal perfusion RFA electrode, could improve the HSV-TK gene transfection, and that RFH treatment at 42 °C with 30 min could further significantly enhance the HSV-TK gene transfection, achieving an excellent therapeutic effect on liver cancer, lung cancer, ovarian cancer, and esophageal cancer in animal models [[77], [78], [79], [80], [81]]. These results demonstrated that sublethal RFH combined with gene therapy may be a promising treatment for some malignant solid tumors. However, further clinical studies are warranted to confirm its efficacy.

## 7 Conclusions and future prospects

Sublethal RFH can enhance the treatment effects of chemotherapy, radiotherapy, immunotherapy, and gene therapy in malignant solid tumor. This notion may present on opportunity to lower the rates of residual and recurrent tumors after RFA for malignant solid tumors. Further studies on its clinical applications are required to confirm this assumption. The disadvantage of this type therapy was that the uneven distribution of heat and therapeutic agents in tumors may affect the treatment effects. However, with the development of a uniformly distributed local drug delivery systems and the improvement of RFA equipment, these issues may be effectively resolved in clinical practice.

Statement of Ethics: This manuscript is a review of published studies and it did not involve ethic issues regarding animals or human beings, so an institutional ethical review and approval were not necessary.

## Funding

This study was supported by the grants of the 10.13039/501100001809National Natural Science Foundation of China (No. 82372069 and No. 82072041) and the Outstanding Youth Science Foundation of Hubei Province, China (2023AFA107).

## Data availability statement

No data was used for this research described in the article.

## CRediT authorship contribution statement

**Jiayun Liu:** Writing – original draft. **Guilin Zhang:** Writing – original draft. **Xinyi Li:** Investigation. **Chuansheng Zheng:** Supervision. **Xuefeng Kan:** Supervision.

## Declaration of competing interest

The authors declare that they have no known competing financial interests or personal relationships that could have appeared to influence the work reported in this paper.
